# Roles of Aa*VeA* on Mycotoxin Production *via* Light in *Alternaria alternata*

**DOI:** 10.3389/fmicb.2022.842268

**Published:** 2022-02-18

**Authors:** Liuqing Wang, Meng Wang, Jian Jiao, Hongmei Liu

**Affiliations:** ^1^Institute of Quality Standard and Testing Technology of BAAFS (Beijing Academy of Agriculture and Forestry Sciences), Beijing, China; ^2^Institutes of Science and Development, Chinese Academy of Sciences, Beijing, China; ^3^Academy of National Food and Strategic Reserves Administration, Beijing, China

**Keywords:** *Alternaria alternata*, mycotoxin, tenuazonic acid, alternariol, light regulation, velvet complex

## Abstract

*Alternaria alternata* is a principal plant pathogen responsible for the biosynthesis of mycotoxins, including tenuazonic acid (TeA), alternariol (AOH), and alternariol monomethyl ether (AME). The velvet gene *VeA* is involved in fungal growth, development, and secondary metabolism, including mycotoxin biosynthesis *via* light regulation. In this study, the detailed regulatory roles of Aa*VeA* in *A. alternata* with various light sources were investigated from the comparative analyses between the wild type and the gene knockout strains. In fungal growth and conidiation, mycelial extension was independent of light regulation in *A. alternata*. Red light favored conidiation, but blue light repressed it. The absence of Aa*VeA* caused the marked reduction of hyphae extension and conidiophore formation even though red light could not induce more spores in ΔAa*VeA* mutant. The differentially expressed genes (DEGs) enriched in hyphal growth and conidiation were drastically transcribed from the comparatively transcriptomic profile between the wild type and ΔAa*VeA* mutant strains with or without light. In mycotoxin production, TeA biosynthesis seems no obvious effect by light regulation, but AOH and AME formation was significantly stimulated by blue light. Nevertheless, the disruption of Aa*VeA* resulted in a marked decrease in mycotoxin production and the action of the stimulation was lost *via* blue light for the abundant accumulation of AOH and AME in the ΔAa*VeA* strain. From DEG expression and further verification by RT-qPCR, the loss of Aa*VeA* caused the discontinuous supply of the substrates for mycotoxin biosynthesis and the drastic decline of biosynthetic gene expression. In addition, pathogenicity depends on Aa*VeA* regulation in tomato infected by *A. alternata in vivo*. These findings provide a distinct understanding of the roles of Aa*VeA* in fungal growth, development, mycotoxin biosynthesis, and pathogenicity in response to various light sources.

## Introduction

The genus *Alternaria* forms colonies worldwide as saprophytes in plant residue, soil, and air, as well as in pathogens of pre- and post-harvest crops (Thomma, 2003). *Alternaria alternata* is the most common species contaminating a wide range of plants, including wheat, sorghum, tomato, apple, and their products (Logrieco et al., 2009), which produces a variety of mycotoxins treating human and animal health. Generally, of the *Alternaria* mycotoxins, tenuazonic acid (TeA), alternariol (AOH), and alternariol monomethyl ether (AME) are the most serious and frequent toxin contaminants (Ostry, 2008; Patriarca, 2016). TeA is the most toxic of *Alternaria* mycotoxins and exhibits acute toxicity and cytotoxicity by suppressing protein biosynthesis on ribosomes and has phytotoxic activity by blocking photosystem II electron transport (Logrieco et al., 2009; Chen and Qiang, 2017). AOH and AME possess cytotoxicity, genotoxic, and mutagenic properties (Patriarca, 2016; Wenderoth et al., 2019). Additionally, AOH also plays as a pathogenicity factor during plant infection (Wenderoth et al., 2019). Furthermore, data on dietary exposure have been recorded to estimate its prevalence in Europe [EFSA on Contaminants in the Food Chain (CONTAM), 2011; Arcella et al., 2016]. However, no regulations have been developed worldwide, except by the Bavarian Health and Food Safety Authority, who set a TeA maximum limit at 500 μg/kg in sorghum/millet-based infant food (Solfrizzo, 2017).

TeA is regarded the hybrid of an isoleucine and two acetates from the skeleton structure. It was identified to be biosynthesized by TeA synthetase 1 (TAS1), a nonclassical type of non-ribosomal peptide synthetase and polyketide synthase (NRPS-PKS) hybrid enzyme (Yun et al., 2015). TAS1 catalyzes cyclization from an isoleucine with an acetoacetyl-CoA. Furthermore, *TAS1* expression is specifically modulated by a Zn(II)2-Cys6-type transcriptional factor encoded by *TAS2*, located on the neighboring *TAS1* in *M. oryzae* (Yun et al., 2017). However, based on the Basic Local Alignment Search Tool (BLAST) results, no homologous proteins (similarity <30%) were found after searching the genome of *A. alternata*. AOH and its methyl derivative AME were confirmed to be biosynthesized by a PKS gene cluster, including *pksI* and *omtI* responsible for their formation (Wenderoth et al., 2019). A Gal4-like transcription factor aohR positively modulated the expression of the gene cluster. In addition, a pksI orthologue SnPKS19 was validated to be responsible for AOH production in the wheat pathogen *Parastagonospora nodorum* (Chooi et al., 2015).

Fungal growth, development, and secondary metabolism are regulated by light signals *via* light sensing systems, including photoreceptor-like white collar (WC-1 and 2) orthologs and phytochrome FphA (Blumenstein et al., 2005; Idnurm and Heitman, 2005). In response to light regulation, the heterotrimeric velvet complex VelB/VeA/LaeA controls secondary metabolites in *Aspergillus nidulans* (Bayram et al., 2008). These components of the velvet complex exert diverse functions in different fungal species. Several scientists have separately characterized the functions of LaeA, VeA, and VelB in mycotoxin producing fungi (Amaike and Keller, 2009; Wiemann et al., 2010; Chang et al., 2013; Estiarte et al., 2016; Takao et al., 2016; Eom et al., 2018). VeA is regarded as an important light regulator, influencing fungal growth, development, and secondary metabolite biosynthesis (Wiemann et al., 2010; Merhej et al., 2012). LaeA was identified as the main regulator of secondary metabolites and as a global regulator for asexual and sexual development, growth, and virulence (Wiemann et al., 2010). PoLAE1 positively regulates TeA biosynthesis *via TAS1* and *TAS2* expression in *M. oryzae* (Yun et al., 2017). Moreover, the regulatory function of LaeA and VeA for AOH and AME production was characterized in a previous study (Estiarte et al., 2016). Both regulators could modulate the expression of genes responsible for their biosynthesis, including *pksJ* and *altR* (Saha et al., 2012; Wenderoth et al., 2019). In addition, growth morphology and conidiation were markedly altered *via* comparative analysis between the wild type and mutant of *LaeA* or *VeA* loss in response to light or dark conditions. However, light was characterized by no obvious differences in AOH and AME formation from the comparison of mycotoxin contents in both strains of *A. alternata* cultured under either dark or light conditions. On the contrary, in the study on the roles of blue-light receptor LreA for mycotoxin production, AOH formation was stimulated by light, especially blue light (Pruß et al., 2014). The mechanism of the contradictive outcomes on the roles of AOH production by light remains unclear from these two studies. Additionally, the effects on the critically important mycotoxin TeA production in response to light have not yet been characterized.

To our knowledge, this is the first report on the roles and potential mechanisms of Aa*VeA* on TeA production with various light sources. In this study, the deletion of Aa*VeA* was carried out, and the analyses of fungal growth, development, and mycotoxin biosynthesis were compared between the wild-type and ΔAaVeA strains under diverse light conditions. Furthermore, the comparatively transcriptomic profile was performed to uncover the regulatory mechanisms for mycotoxin production. Blue light was unfavorable to sporulation but did not obviously affect hyphal extension in *A. alternata*. In contrast, red light had no significant effect on sporulation, whose *in vitro* morphology was similar to that of white-light treatment. TeA biosynthesis depended on the regulation of AaVeA, but light caused no marked alteration on its production, which was distinct from the stimulation of AOH and AME biosynthesis by blue light.

## Materials and Methods

### Strains and Culture Conditions

*A. alternata* P3 and the mutant strains were cultured on potato dextrose agar (PDA) at 25°C. The spores were then washed with 0.1% Tween-80 water, collected from the culture of *A. alternata*, and diluted to 1 × 10^5^ spores/ml (Jiang et al., 2019; Wang et al., 2019). The spore suspension was cultured on PDA at 25°C for 10 days to monitor hyphal extension, colony morphology, and conidial formation (Wang et al., 2019). The spores were inoculated and incubated on potato dextrose broth (PDB) under dark, white, blue, and red-light conditions at 25°C for 10 days for the following analysis on mycotoxin determination and gene expression. For the continuous light experiment, white, blue, or red LED lamps (18 W/lamp) were used, and the distance between the light source and the medium was 30 cm. The average illumination intensities of white, blue, and red light were separately measured at 5200, 4350, and 2,270 lux, respectively.

### Construction of the Aa*VeA* Knockout Mutant

The deletion of Aa*VeA* was used to investigate the functional roles for the growth, development, and secondary metabolite biosynthesis. Aa*VeA* was disrupted by homologous recombination using the hygromycin resistance gene as a marker gene for antibiotic resistance screening (Supplementary Figure S1). Flanking sequences of 1,458-bp upstream and 1,485-bp downstream were separately amplified by polymerase chain reaction (PCR) using genomic DNA of *A. alternata* P3 as a template with the primer pairs Aa*VeA*-up-F/R and Aa*VeA*-down-F/R (Supplementary Table S1). The hygromycin resistance gene encoding **h**ygromycin **ph**osphotransferase (hph) was amplified using the primer pair *hph*-F/R. The reaction mixture (50 μl) consisted of 2 μl of the template DNA, 1 μl of each forward and backward primer (10 μM), 25 μl of GoTaq Master Mix (Promega, Madison, WI, United States), and 21 μl of nuclease-free water. The PCR program was set as follows: initial denaturation at 95°C for 5 min, 35 cycles of the amplification at 95°C for 30 s, 54°C for 30 s and 72°C for 90 s, and finally extension for 5 min. The PCR product was extracted using a Gel Extraction Kit (Sangon Biotech, Shanghai, China). Three fragments were assembled by overlapping extension PCR in combination with nested PCR using the primer pair Aa*VeA*-knock-F/R. The assembled fragment was also purified for transformation. The protoplast of *A. alternata* was obtained by enzymatic hydrolysis of fungal cell wall with 30 mg/ml of snailase, 5 mg/ml of driselase, 2 mg/ml of lyticase, 8 mg/ml of β-D-dextranase, and 20 mg/ml of cellulase. Then, the PCR product was transformed into the protoplast by polyethylene glycol (PEG)-CaCl_2_ mediated genetic transformation. The transformants were screened by 100 μg/ml hygromycin B (Roche Diagnostics, Mannheim, Germany) and identified by PCR and quantitative PCR according to the method of Zhang et al. (2016). The mutant of Aa*VeA* deletion was finally obtained for the following study (Supplementary Figure S1).

### Hyphal Growth, Conidial Formation, and Microscopic Observation

The spore suspension of *A. alternata* P3 and the mutant was acquired (1 × 10^5^ spores/ml). A 5-μl aliquot of the suspension was inoculated in a PDA medium and grown for 10 days at 25°C. The characteristics of the colony were observed, and then the conidiation was compared between the wild type and ΔAa*VeA* mutant under white, red, blue, and dark conditions. The spore counts were determined using the hemocytometer after they were washed completely from the culture using 0.1% Tween-80.

The microscopical structure was evaluated by scanning electron microscopy (SEM). The samples were harvested and fixed in 2.5% glutaraldehyde. Then, the samples were washed, dehydrated, and vacuum-dried following the processes by Wang et al. (2019).

### Mycotoxin Detection

The 1%(v/v) spore suspension of *A. alternata* P3 and the Aa*VeA* null mutant were inoculated and cultured in PDB. The strains were incubated for 10 days at 25°C with or without various light treatments. The supernatant was centrifuged and used to determine the concentration of mycotoxin. The determination of mycotoxin production was assayed under the direction of Jiang et al. (2019) using LC–MS/MS.

### Transcriptomic Analyses

The mycelial samples were collected after culture under various light conditions and ground with liquid nitrogen. Total RNA was extracted from the mycelia with three biological replications cultivated in PDB medium with or without light using TRIzol reagent according to the Cold Spring Harbor Laboratory (Rio et al., 2010). The quality and purity of the extracted RNA were evaluated using the Bioanalyzer 2,100 and RNA 6000 Nano LabChip Kit (Agilent, CA, United States) with RIN number > 7.0. cDNA library construction and RNA-seq were carried out by Hangzhou Lianchuan Biotechnology Co., Ltd. (Hangzhou, China). Using the Illumina paired-end RNA-seq approach, the transcriptome was sequenced, and millions of paired-end reads were generated. After removing the low-quality reads, we mapped the reads to the reference genome of *A. alternata* using the HISAT package (Kim et al., 2015). HISAT allows multiple alignments per read (up to 20 by default) and a maximum of two mismatches when mapping the reads to the reference. The remaining reads, after trimmed from the raw reads, were then bioinformatically analyzed based on the genome annotation of *A. alternata* (genome assembly: GCF_001642055.1; Dang et al., 2015).

Differentially expressed genes (DEGs) were selected with a |log2 (fold change) | > 1 and statistical significance of *value of p* < 0.05 by the R package. Gene Ontology (GO) functional annotation (Young et al., 2010) and Kyoto Encyclopedia of Genes and Genomes (KEGG; Kanehisa et al., 2008) pathway enrichment analysis were conducted with the DEGs to further understand their functions.

### Confirmation of Gene Relative Expression

The relative expression levels of TeA biosynthetic genes and velvet genes were further confirmed and quantified by reverse transcription quantitative real-time PCR (RT-qPCR). Total RNA was separately extracted by the EasyPure Plant RNA Kit (TransGen Biotech, Beijing, China) containing DNase I digestion for the genomic DNA residue. The RNA samples were diluted to an equal concentration, and then cDNA was reverse transcribed by EasyScript One-Step gDNA Removal and cDNA Synthesis SuperMix (TransGen Biotech, Beijing, China). Quantitative PCR was carried out by TransStart Top Green qPCR SuperMix (+Dye I), using cDNA as the template. The 20-μL reaction mixture comprised the following: 2 μl of cDNA, 0.4 μl of forward and backward primer (10 μM; Supplementary Table S2), 10 of 2 × TransStart Top Green qPCR SuperMix (+Dye I), and 7.2 μl of nuclease-free water. The transcriptomic level of clustered biosynthetic and regulatory genes was separately normalized to the expression of β-tubulin and evaluated by the 2^-ΔΔCt^ calculation from the results of StepOnePlus Real-Time PCR Systems (Applied Biosystems, Foster City, CA, United States).

### Fungal Infection on Tomato

To evaluate the pathogenicity and mycelial extension *in vivo* for the wild and VeA-disrupted strains of *A. alternata*, an equal volume (5 μl) of conidial suspension (10^5^/ml) was separately inoculated into the man-made wound on the surface of tomatoes after they were sterilized with 10% sodium hypochlorite and 75% alcohol. Tomatoes were treated with or without light at 25°C for 10 days.

Fungal growth was measured, and the infected area on tomatoes was used to extract and determine mycotoxin production. The samples were first dried by vacuum freezing and drying technology. Then, they were weighed and ground into powder, of which 0.2 g was used to determine the mycotoxin content. The final mycotoxin concentration was expressed as mycotoxin content per unit area.

### Statistical Analysis

All the experiments were carried out with at least three replicates. The data of fungal extension diameters, spore counts, and mycotoxins concentrations were analyzed by IBM SPSS statistics 23.0 (IBM Inc., Armonk, NY, United States) and compared by the one-way analysis of variance (ANOVA) using Tukey’s test. Significance was considered as *p* < 0.05. The figures were generated by Graphpad Prism 8.02 (GraphPad Software Inc., San Diego, CA, United States).

## Results

### Blue Light Inhibits Spore Formation

**Figure 1:**
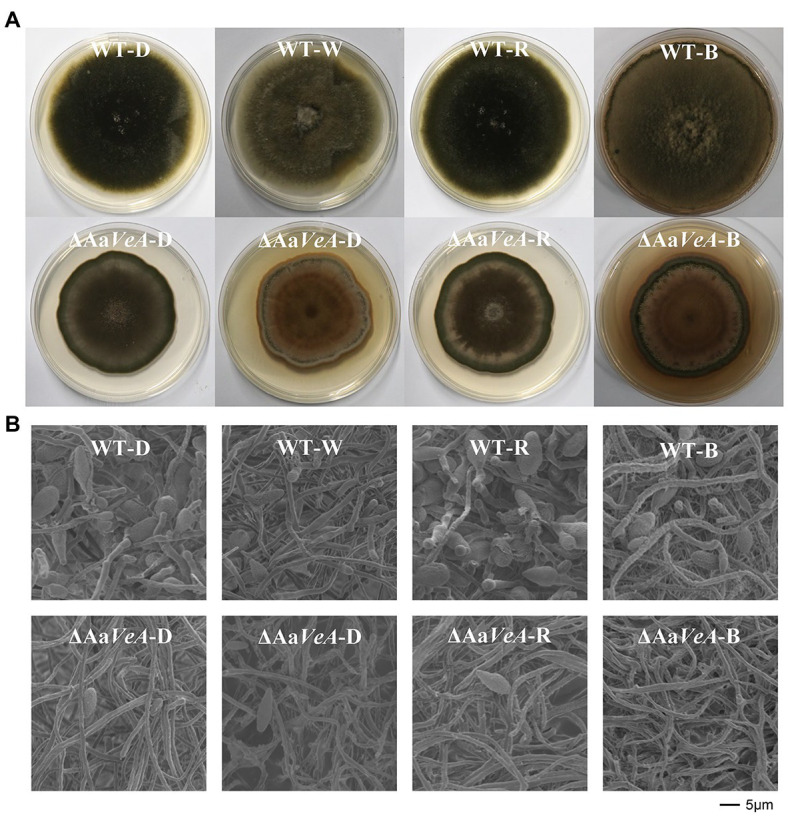
Colony and morphology characters of the wild type (WT) and Aa*VeA* disrupted mutant (ΔAa*VeA*) strains with various light sources in *Alternaria alternata*. **(A)** The front diagram of WT and ΔAa*VeA* strains cultured on potato dextrose agar (PDA) for 10 days at 25°C in darkness (D) or with white (W), red (R) and blue (B) light. The colony of the wild strain extended at a similar rate while the mutant strain grew slower. The similar morphology was observed in the colony of the wild strain in darkness and with red light. However, the density of the mycelia became thicker in the wild strain with white and blue light. **(B)** Microscopic morphology of mycelia and spores of WT and ΔAa*VeA* strains by scanning electron microscopy (SEM). The microscopic morphology of mycelial and spore structure was observed with no marked alteration in dark and light conditions. However, the fungal spores were much more abundant under dark and red-light conditions.

**Figure 2:**
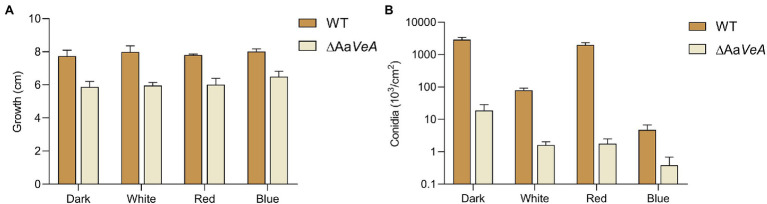
Mycelial growth **(A)** and conidia formation **(B)** of WT and ΔAa*VeA* strains. The colony diameter of WT and ΔAa*VeA* strains cultured on PDA medium was separately measured. Accordingly, the conidia were washed and determined using the hemocytometer.

To understand the effects of the Aa*VeA* gene on fungal growth and conidiation, the wild type and Aa*VeA* knockout strains were used to characterize the properties with white, red, or blue light or without light. The wild type and the ΔAa*VeA* mutant strain were separately grown on PDA for 10 days. The colonies and the microscopic morphology are shown in Figure 1. Light seemed to hardly influence the mycelial extension of *A. alternata* from the colonies with light illumination (Figure 2A). However, lacking the gene Aa*VeA* resulted in the inhibition of hyphal growth by 24.2% in the ΔAa*VeA* mutant under dark conditions (*p* < 0.001). Similarly, the mycelia of the mutant extended 25.5%, 23.0%, and 18.9% more slowly than the wild type with white, red, or blue light, respectively. There was no significant difference in the inhibition rate under the different light conditions due to the gene deletion (*p* > 0.05).

Although no obvious difference and a similar trend was found on the hyphal extension, the conidiation of *A. alternata* was markedly changed under different conditions (Figures 1B, 2B). White light illumination caused a 97.2% decrease in spores (*p* < 0.001). Red light stimulated conidiation, while blue light had a negative effect on conidial formation. Compared to the dark condition, red light recovered about 68.7% of conidial formation, but under blue light, the formation of conidia was repressed by 99.8%. Different from mycelial extension, the disruption of Aa*VeA* led to a significant reduction in conidia under both dark and light conditions. Interestingly, unlike the wild strain, red light hardly stimulated asexual sporulation when the Aa*VeA* gene was inactivated. Blue light also resulted in the lack of sporulation in both wild and ΔAa*VeA* strains.

In general, light hardly affected fungal growth, while red light favored spore formation and blue light did the opposite.

### Mycotoxin Production Depends on Aa*VeA* Regulation

**Figure 3:**
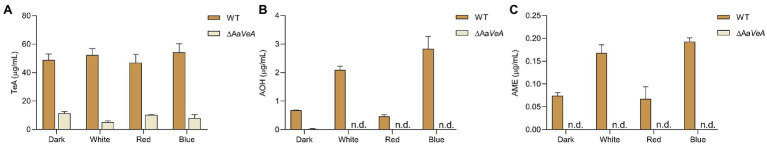
Mycotoxin biosynthesis produced by the WT and Aa*VeA* disrupted mutant (ΔAa*VeA*) strain of *Alternaria alternata* under various light sources. The concentrations of mycotoxins, including tenuazonic acid (TeA; **A**), alternariol (AOH; **B**), and alternariol monomethyl ether (AME; **C**), were, respectively, extracted and determined by LC–MS.

To evaluate the Aa*VeA* roles in TeA biosynthesis, mycotoxin extraction and quantitation were performed, and the changes in mycotoxin production were compared between the wild-type and ΔAa*VeA* strains under various light sources. The TeA concentration reached up to 48.8 μg/ml in darkness, and no significant change was observed with white light illumination (Figure 3A). Furthermore, mycotoxin production also seemed no more marked fluctuation under red and blue light conditions. TeA production became much weaker after the gene, Aa*VeA*, was disrupted under dark or light conditions. Similarly, no obviously positive or negative effect on TeA biosynthesis was detected in the mutant strain in response to various light sources (*p* > 0.05).

In a previous study, light promoted AOH and AME production (Pruß et al., 2014; Igbalajobi et al., 2019), which was also confirmed in this study (Figures 3B,C), although it could not result in more accumulation in TeA biosynthesis. AOH and AME concentrations were extremely stimulated by light illumination, especially blue light. AOH and AME contents were separately promoted 2.08- and 1.26-times with white light higher than its production in the dark (*p* < 0.001). Furthermore, blue light exposure caused much more AOH and AME accumulation while red light had no positive stimulation. When Aa*VeA* was deleted, AOH and AME production was significantly repressed by 96.4% and 100% in the dark (*p* < 0.001). Light could no longer stimulate more abundant AOH and AME biosynthesis, even blue light.

### Transcriptomic Response Under Aa*VeA*

To uncover the regulatory roles of Aa*VeA*, the detailed transcriptomic profile of *A. alternata* with or without light illumination was separately analyzed and shown in Supplementary Tables S3 and S4. In total, gene expression at the transcriptional level exhibited the most dramatic changes from the number of DEGs in wild type strain compared to the ΔAa*VeA* mutant grown in the dark (Supplementary Figure S2). There were 1,496 DEGs, including 614 upregulated and 882 downregulated DEGs. Nevertheless, the comparative transcriptomic profile of the mutant had fewer fluctuations between the dark and white light conditions, as there were 660 DEGs in total (350 upregulated and 310 downregulated genes). This demonstrated that Aa*VeA* is a critically important regulator for the physiological-biochemical processes in *A. alternata*.

**Figure 4:**
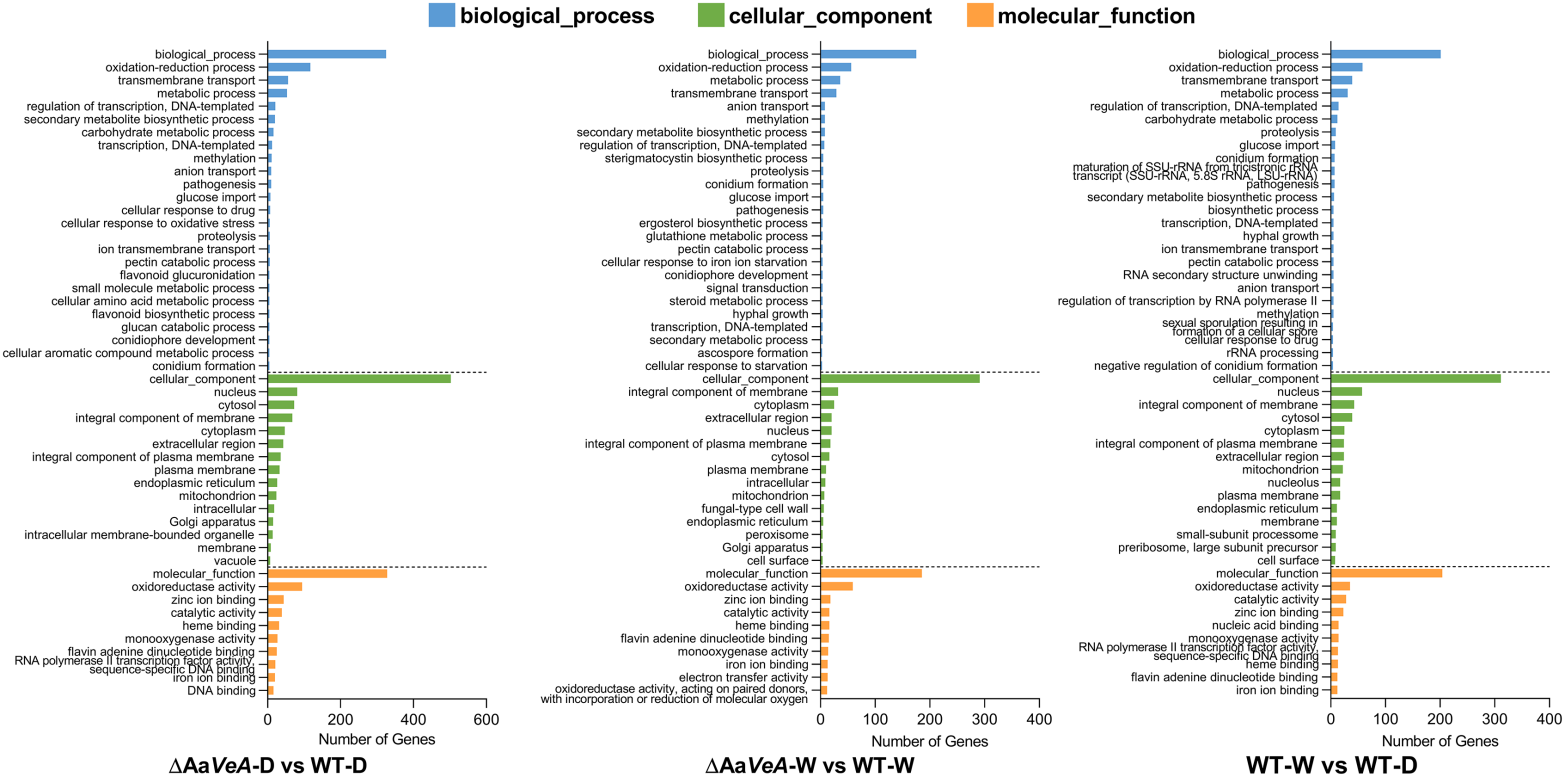
Gene Ontology (GO) enrichment analysis of differentially expressed genes (DEGs). The comparative DEGs were employed to carry out GO analysis in both groups of ΔAa*VeA*-D/WT-D (darkness), ΔAa*VeA*-W/WT-W (white light illumination), and WT-D/WT-W. The number of distribution of DEGs in GO terms enriched in biological process, cellular component, and molecular function was reflected as the histogram.

**Figure 5:**
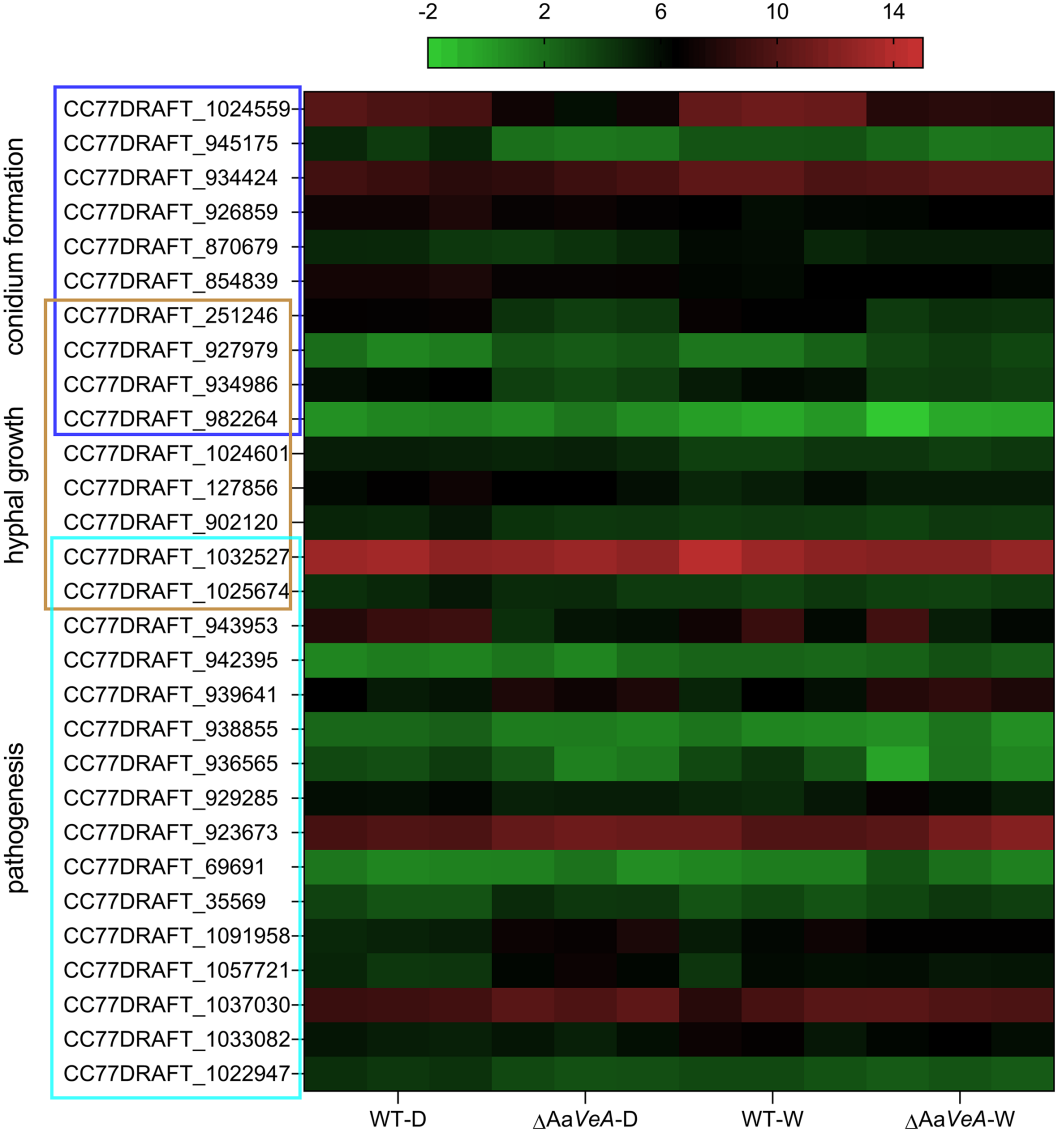
The heat map of the transcriptional level of DEGs enriched in the biological process of hyphal growth, conidium formation, and pathogenesis. The transcriptional level of DEGs was expressed as the value of log_2_ FPKM (Fragments Per Kilobase per Million) from the detailed transcriptomic profile.

From the analysis of GO enrichment, some biological processes, including conidium formation, pathogenicity, and secondary metabolite biosynthetic process, were distinctly enriched from the comparatively expressed DEGs (Figure 4). The biological process related to conidial formation and development was enriched from the DEGs between the wild and Aa*VeA* null strains under white light and dark conditions (Figure 5). The secondary metabolite biosynthetic process was markedly influenced by Aa*VeA* regulation. The biosynthesis of mycotoxins, including TeA, AOH, and AME, were affected to various degrees under light regulation *via* the regulator.

Many DEGs partly enriched in the process of hyphal growth (Figure 5). This explains why the mycelial extension became slower when Aa*VeA* was disrupted in *A. alternata*. In addition, the DEGs responsible for pathogenesis were enriched and hence Aa*VeA* was probaly involved in the pathogenicity of fungal infection. To investigate whether Aa*VeA* was responsible for pathogenicity, the trials were carried out in tomato infected by *A. alternata* with or without Aa*VeA*.

From KEGG pathway enrichment analysis, the metabolism, biosynthesis, and degradation of amino acids, including valine, leucine, and isoleucine were found to be enriched (Supplementary Figure S3). The substrate of TeA biosynthesis comprises isoleucine and acetoacetyl-CoA. The fluctuation of gene expression related to amino acid metabolism could lead to the alteration of the supply of isoleucine. Acetoacetyl-CoA could be formed by carbohydrate metabolism, including fatty acid biosynthesis and degradation, which were also enriched in KEGG pathway enrichment analysis.

### Effect of Aa*VeA* on the Expression of Mycotoxin Biosynthetic and Regulatory Genes

**Figure 6:**
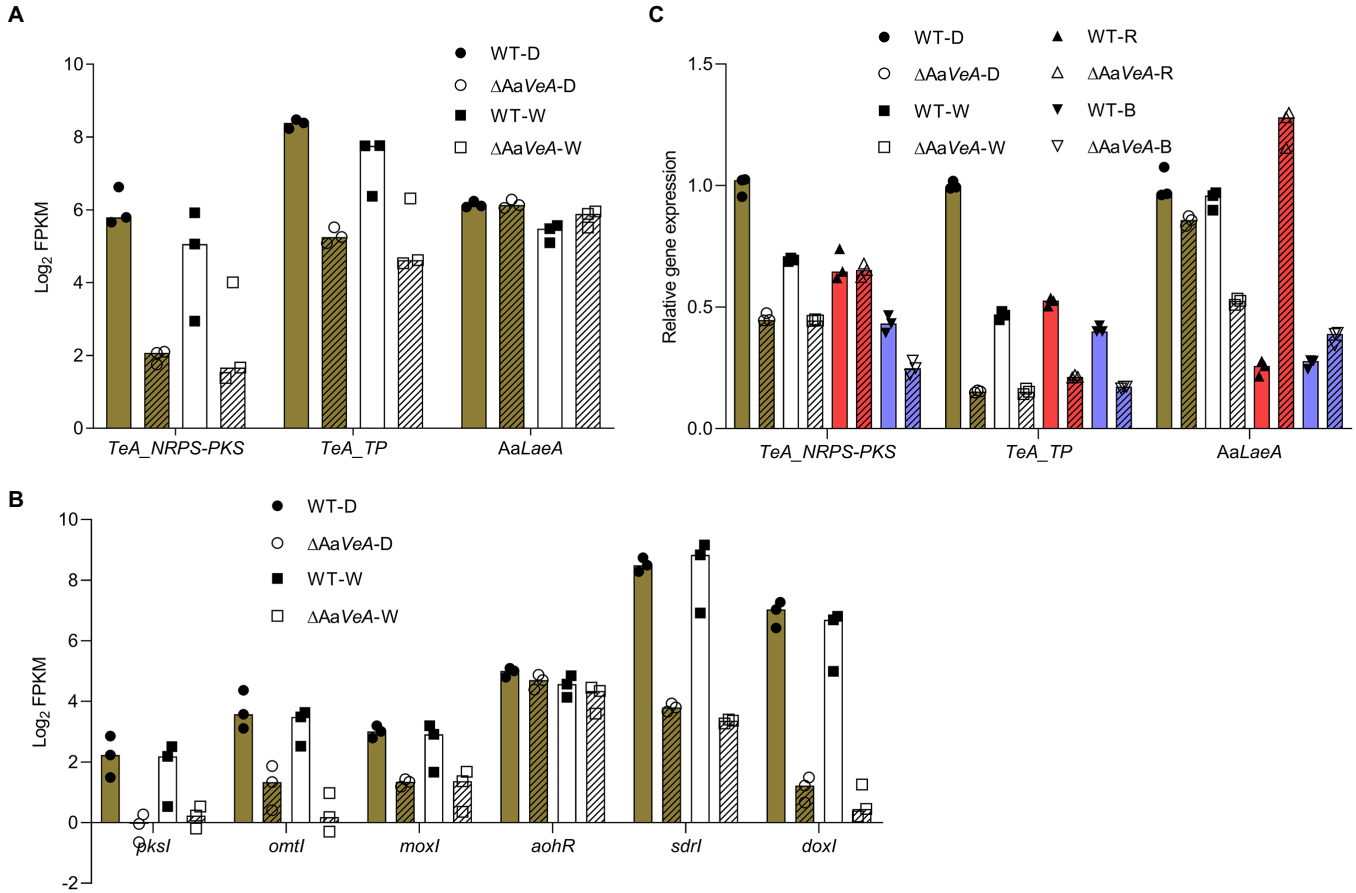
The expression analyses of biosynthetic and regulatory genes involved in mycotoxin biosynthesis. The biosynthetic and regulatory genes contain the biosynthetic clustered genes of TeA and AOH/AME, and the master regulator gene of secondary metabolites Aa*LaeA* in *Alternaria alternata*. The results of the gene expression analyses were plotted using the median with range and separately expressed as the scatter dots. **(A)** The expression level of TeA biosynthetic clustered genes and Aa*LaeA* was expressed as the value of log_2_ FPKM from the transcriptomic analysis. **(B)** The expression level of biosynthetic clustered genes for AOH and AME biosynthesis was expressed as the value of log_2_ FPKM from the transcriptomic analysis. **(C)** The relative gene expression was confirmed by reverse transcription quantitative real-time PCR (RT-qPCR) and calculated by the 2^-ΔΔCt^ method.

From the prediction of the secondary metabolite biosynthesis gene clusters, the TeA biosynthetic gene cluster contains two genes encoding a biosynthetic enzyme and a transporter. The enzymatic protein has a NRPS portion and a ketosynthase (KS) domain for the condensation and cyclization of the mycotoxin, which is similar to the homologous protein encoded by the *TAS1* (*MGG_07803*) gene in *Magnaporthe oryzae* (Yun et al., 2015). Additionally, another *TAS2* gene encoding a Zn(II)2-Cys6-type transcription factor harbored the neighboring *TAS1* and was identified to be responsible for the regulation of *TAS1* expression and mycotoxin biosynthesis (Yun et al., 2017). However, we searched for the homologous gene throughout the genome of *A. alternata*, but no matched protein was found. The transporter gene may be responsible for the export of TeA from the cytoplasm to the extracellular matrix. Generally, the trend of these two genes expression was similar to the production of the mycotoxin (Figure 6A). Accordingly, AOH/AME biosynthetic gene cluster has been identified, which consists of four biosynthetic enzymatic genes (*pksI*, *omtI*, *moxI*, *sdrI*, and *doxI*) and a transcriptional factor gene (*aohR*; Chooi et al., 2015; Wenderoth et al., 2019). Among them, *pksI* is responsible for AOH formation. AOH is then converted to AME by an O-methyltransferase encoded by *omtI*. Both the transcription of *pksI* and *omtI* became lower but the Aa*LaeA* gene transcript had no downtrend in the Aa*VeA* knockout strain (Figure 6B). This demonstrates that Aa*VeA* could positively affect TeA biosynthesis by regulating the transcription of the clustered biosynthetic genes, but it did not modulate Aa*LaeA* expression.

To validate the data of RNA-seq, RT-qPCR was carried out for the analysis of fungal gene expression (Figure 6C). The trend of the clustered genes and *LaeA* transcription was similar to the analysis from RNA-seq. In addition, the red and blue light treatments had similar effects in the wild type and ΔAa*VeA* strains.

### Aa*VeA* Role in the Pathogenicity of *Alternaria alternata*

**Figure 7:**
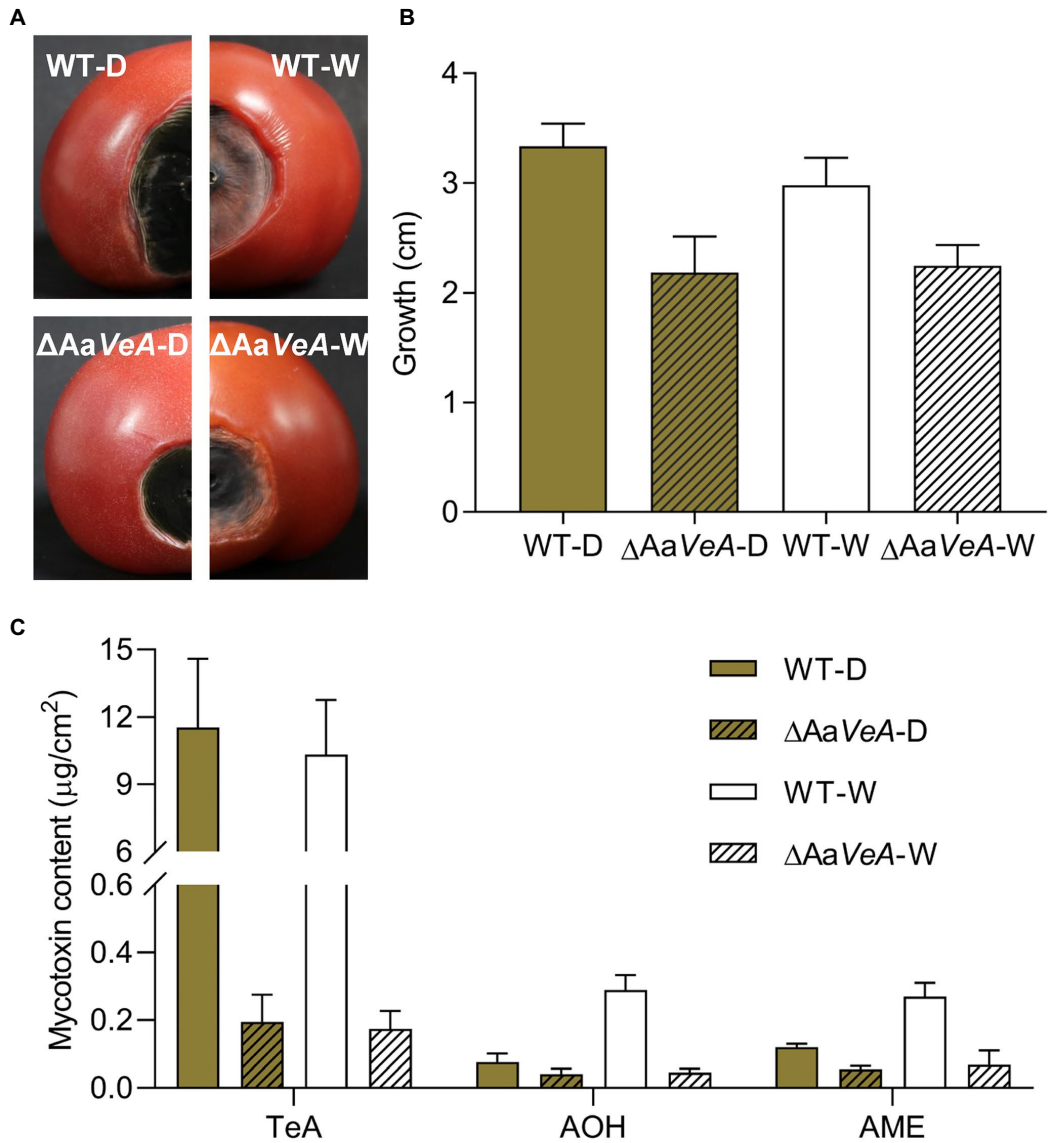
The infection and mycotoxin production of ΔAa*VeA* compared to the wild type (WT) strain on tomatoes with or without light treatment. The spore suspension of WT and ΔAa*VeA* strains was separately inoculated onto tomatoes (1 × 10^5^ spores/ml; 5 μl) and cultured for 10 days at 25°C under dark or white light condition. **(A)** The diagram of tomatoes infected by WT and ΔAa*VeA* strains of *Alternaria alternata*. **(B)** The growth diameter of fungal infection. **(C)** The mycotoxin production of *Alternaria alternata* on the tomatoes.

The wild and ΔAa*VeA* mutant strains of *A. alternata* were separately used to inoculate and colonize the surface of sterilized tomatoes. For 10 days culture, the disruption of Aa*VeA* resulted in a reduction of 34.6% or 24.7% in lesion size under dark or light conditions, respectively (Figure 7). The hyphae of the mutant had weakness to be extending. Light seemed to have no significant influence on hyphal growth in the wild strain, which was consistent with the results of medium culture.

Light had no obvious influence as an activator or inhibitor for TeA biosynthesis. Accordingly, TeA production was obviously decreased by 98.3% in the ΔAa*VeA* strain grown in the dark (*p* < 0.001). A similar reduction occurred under white light illumination. However, light stimulated the accumulation of AOH and AME by 2.81- and 1.24-times more than that of mycotoxin production in the dark (*p* < 0.001). Moreover, AOH and AME contents were separately suppressed by 45.9% and 53.7%, respectively, due to the deletion of Aa*VeA* under dark condition. Similarly, the loss of Aa*VeA* led to a decrease in the AOH and AME concentration by 84.3% and 74.7% in ΔAa*VeA* with light illumination. This showed that AOH and AME biosynthesis was regulated by light *via* Aa*VeA*.

In total, these results from the pathogenic infection on tomato *in vivo* were similar to the outcome *in vitro*, in which Aa*VeA* positively played vital roles in hyphal growth, pathogenicity, and mycotoxin biosynthesis.

## Discussion

Light modulates fungal growth, development, and virulence (Idnurm and Heitman, 2005; Purschwitz et al., 2006; Losi and Gaertner, 2021). The mode of action of various filamentous fungi has different responses under light conditions (Losi and Gaertner, 2021). Members of the fungal kingdom have developed light sensing systems, such as phytochrome FphA as a red-light photosensor (Blumenstein et al., 2005) and white collar (WC-1 and 2) homologs as blue-light photoreceptors (Idnurm and Heitman, 2005). Accordingly, FphA has been suggested positive function for sporulation while LreA, the WC-1 orthologue, is required for the spore formation independently of light in *A. alternata* (Pruß et al., 2014; Igbalajobi et al., 2019). The velvet complex, including VeA, LaeA, and VelB proteins, has been found to be involved in fungal growth, development, and virulence in response to light (Bayram et al., 2008; Amaike and Keller, 2009). In this study, the disruption of Aa*VeA* enabled weaker growth and pathogenesis independent of light stimulation in *A. alternata*. Red light stimulates conidiospore formation, and blue light represses it, which is consistent with a previous study, in which blue light acted as a suppressor in *A. alternata* and *A. cichorii* (Vakalounakis and Christias, 1985, 1986; Pruß et al., 2014). Nevertheless, continuous light favors sporulation in *A. eichhorniae* (Shabana et al., 2000). The distinct results for conidiation in light conditions may be due to the diversity of species in the genus *Alternaria* and culture conditions (Varma et al., 2015). The loss of Aa*VeA* results in the marked reduction of spore formation in *A. alternata*. Even red light could not stimulate more spores in the mutant strain and thus Aa*VeA* was required for sporulation.

Light modulation could affect fungal metabolism as well, including primary and secondary metabolism (Tisch and Schmoll, 2010; Fanelli et al., 2015). Light regulates fungal primary metabolism, such as the production of carotenoids in the previous studies in the genus *Fusarium* (Estrada and Avalos, 2008; Tang et al., 2020), as well as *Neurospora crassa* (Nelson et al., 1989). In addition to the influence of primary metabolites, light was confirmed to be an important signal for secondary metabolism, including mycotoxin production (Fanelli et al., 2015). Furthermore, the effects of light were distinctly different on the biosynthesis of various secondary metabolites. For instance, light played no significant role in penicillin production in *Penicillium chrysogenum* (Kopke et al., 2013). Unlike *P. chrysogenum*, penicillin biosynthesis seemed to be positively influenced by light in *A. nidulans* (Kato et al., 2003; Spröte and Brakhage, 2007; Röhrig et al., 2017). In the research of mycotoxins, the production of aflatoxin B_1_ by *A. flavus* and ochratoxin A by *A. ochraceus* was considerably higher with light stimulation (Aziz and Moussa, 1997). However, in other reports, light exposure, especially blue light, led to the drastic reduction of ochratoxin A formation in the producing *Penicillium* and *Aspergillus* species (Schmidt-Heydt et al., 2011, 2012). In addition, sterigmatocystin production levels were differently altered under dark or light conditions in *A. nidulans* (Kato et al., 2003; Bayram et al., 2010; Röhrig et al., 2017). Sterigmatocystin biosynthesis was stimulated with the alteration from light to darkness in *A. nidulans* FGSC A4 (Kato et al., 2003; Bayram et al., 2010). Nevertheless, Light illumination preferred to the elevation of sterigmatocystin level in *A. nidulans* SRJ7 (Röhrig et al., 2017). In *A. alternata*, light was suggested distinct roles in AOH production in the previous studies (Pruß et al., 2014; Estiarte et al., 2016; Igbalajobi et al., 2019). Hence, we further performed the function of various light sources on the production of AOH and AME as well as TeA. In our study, AOH and AME biosynthesis were confirmed to be stimulated by light, especially blue light. However, TeA production was independently of light in that no stimulative impact occurred with light illumination. This demonstrates the distinct roles in the biosynthesis of various secondary metabolites in response to various light sources.

The heterotrimeric velvet complex, including VeA, LaeA, and VelB, is involved in secondary metabolism by coordinating light signals. In the velvet complex, VeA plays important roles in fungal growth and secondary metabolism in response to various light sources (Wiemann et al., 2010; Merhej et al., 2012; Eom et al., 2018). LaeA is responsible for the biosynthesis of secondary metabolites as the master regulator (Perrin et al., 2007; Bayram et al., 2008; Kosalková et al., 2009). Interestingly, in the study of the velvet complex for penicillin biosynthesis, veA exhibited opposing roles in penicillin biosynthesis in *A. nidulans* (Kato et al., 2003; Spröte and Brakhage, 2007). Nevertheless, VeA acts similar role as a positive regulator for sterigmatocystin production in *A. nidulans* (Kato et al., 2003; Bayram et al., 2010). The function of LaeA and VeA on AOH and AME production had been performed with or without white light in *A. alternata* (Estiarte et al., 2016). Based on the previous studies, the regulatory impacts of VeA were carried out for the production of AOH, AME, and TeA in *A. alternata* in response to various light sources. The marked reduction of mycotoxins occurred, and light had no stimulative impact on AOH and AME biosynthesis in the Aa*VeA* null strain. Thus, all the mycotoxins were partly regulated by the velvet gene, Aa*VeA*.

Mycotoxins are involved in fungal pathogenicity, as they facilitate mycotoxin-producing fungi to colonize plant tissues or utilize their nutrients (Graf et al., 2012; Wenderoth et al., 2019). TeA exerts phytotoxic activity and induces plant necrosis by blocking electron transport (Chen and Qiang, 2017). In addition, AOH was confirmed as a virulence factor for its contribution to the infection and extension of fungal hosts. In this study, the deletion of Aa*VeA* led to the marked reduction of TeA, AOH, and AME. Therefore, the infection of the mutant became weaker on tomato. In total, this above illustrates that the regulator AaVeA coordinates light signals for mycelial growth, sporulation, pathogenesis, and mycotoxin production.

The continuous supply of substrates is a prerequisite of mycotoxin biosynthesis. Accordingly, TeA has been confirmed to be formed by the condensation of an isoleucine and an acetoacetyl-CoA (Yun et al., 2015). The biosynthesis of AOH and AME initially starts from acetyl-CoA (Saha et al., 2012; Wenderoth et al., 2019). Hence, the influence of metabolic processes may lead to an obvious change in mycotoxin biosynthesis. The loss of Aa*VeA* could cause an obvious reduction in mycotoxin production possibly due to the drastic fluctuation in amino acid metabolic process, carbohydrate metabolic process, secondary metabolites biosynthetic process, etc., which were enriched in the comparatively transcriptomic profile of wild type and Aa*VeA* null strains with or without light.

Generally, secondary metabolites of biosynthetic genes are clustered and harbored closely in mold. Not only TeA but also AOH and AME have been identified. The TeA biosynthetic gene cluster contains at least one enzyme catalyzing the cyclization reaction and a pathway-specific Zn(II)2-Cys6-type transcriptional factor in *M. oryzae* (Yun et al., 2015, 2017). In fact, there is a homologous enzyme responsible for the catalytic reaction, but no specific regulatory gene is harbored near the enzymatic gene. However, there is a neighboring transporter gene, which may be connected to the export of the mycotoxin avoiding toxicity to it. AOH and AME were biosynthesized by a gene cluster consisting of a polyketide synthase gene (*pksI*), an O-methyltransferase (*omtI*), and other enzymatic genes, as well as a transcriptional factor gene, *aohR* (Wenderoth et al., 2019). The disruption of Aa*VeA* led to a drastic decline on the expression level of biosynthetic genes in *A. alternata* under both light and dark conditions, which agrees with the trend of mycotoxin production.

In conclusion, light stimulation had no marked alteration on mycelial growth but caused significant changes in sporulation in *A. alternata*. Although red light favored the formation of spores, blue light weakened the sporulation. However, the lack of Aa*VeA* led to slower fungal growth and the marked reduction of conidiospore formation in *A. alternata* despite light stimulation. The deletion of Aa*VeA* resulted in drastic transcriptional fluctuation of DEGs enriched in hyphal growth, conidiation, and pathogenicity in the wild type and ΔAa*VeA* strains under dark and light conditions. Light, especially blue light stimulated AOH and AME accumulation but had no obvious effects on TeA production. The biosynthesis of these mycotoxins was dependent on the modulation of the velvet gene, Aa*VeA*, in *A. alternata*. The regulator positively modulated mycotoxin production *via* the continuous supply of substrates and the activation of biosynthetic gene expression. From this study, the application of visible light on the prevention and control of the mycotoxins seems inappropriate due to the stimulus for AOH and AME biosynthesis by light, especially blue light. In combination with our previous study, UV-C irradiation could probably be a promising tactic for the mycotoxin control. In addition, it would be more effective to avoid the continuously visual light when some other control measures are taken for reducing the mycotoxins contamination in agricultural products.

## Data Availability Statement

The original contributions presented in the study are publicly available. This data can be found at: NGDC, CRA005946, https://ngdc.cncb.ac.cn/gsa/browse/CRA005946.

## Author Contributions

LW and MW conceptualized and designed the manuscript and wrote and revised the manuscript. LW carried out the experiments. HL and JJ gave the technical supports and revised the manuscript. All authors read and approved the manuscript.

## Funding

This research was supported by the National Natural Science Foundation of China (grant no. 31801648), the National Project for Quality and Safety Risk Assessment of Agricultural Products of China (grant no. GJFP2019002), and the Beijing Natural Science Foundation (grant nos. 6184038 and 7192026).

## Conflict of Interest

The authors declare that the research was conducted in the absence of any commercial or financial relationships that could be construed as a potential conflict of interest.

## Publisher’s Note

All claims expressed in this article are solely those of the authors and do not necessarily represent those of their affiliated organizations, or those of the publisher, the editors and the reviewers. Any product that may be evaluated in this article, or claim that may be made by its manufacturer, is not guaranteed or endorsed by the publisher.

## Abbreviations

TeA: Tenuazonic acid
AOH: Alternariol
AME: Alternariol monomethyl ether
TAS1: TeA synthetase 1
NRPS: Non-ribosomal peptide synthetase
PKS: Polyketide synthase
BLAST: Basic Local Alignment Search Tool
WC: White collar
PDA: Potato dextrose agar
PDB: Potato dextrose broth
PCR: Polymerase chain reaction
Hph: Hygromycin phosphotransferase
PEG: Polyethylene glycol
SEM: Scanning electron microscopy
DEGs: Differentially expressed genes
GO: Gene Ontology
KEGG: Kyoto Encyclopedia of Genes and Genomes
RT-qPCR: Reverse transcription quantitative real-time PCR
ANOVA: Analysis of variance
KS: Ketosynthase
