# Supplementary material for: Roles of AaVeA on Mycotoxin Production via Light in Alternaria alternata
Source: Front Microbiol. 2022 Feb 18;13:842268. doi: 10.3389/fmicb.2022.842268 (PMC8894881; doi:10.3389/fmicb.2022.842268)
Supplement: Supplementary file 2 [file Data_Sheet_1.docx]

**Roles of Aa*VeA* on mycotoxin production via light in *Alternaria alternata***

**Liuqing Wang^1^, Meng Wang^1*^, Jian Jiao^2^, Hongmei Liu^3^**

^1^ Institute of Quality Standard and Testing Technology of BAAFS (Beijing Academy of Agriculture and Forestry Sciences), No. 9 Middle Road of Shuguanghuayuan, Haidian District, Beijing 100097, China

^2^ Institutes of Science and Development, Chinese Academy of Science, No. 15 Zhongguancun North Road, Haidian District, Beijing 100190, China

^3^ Academy of National Food and Strategic Reserves Administration, No.11 Baiwanzhuang Str, Xicheng District, Beijing 100037, China

*** Correspondence:**

Meng Wang

wangm@brcast.org.cn

**Supplementary Table S1** Primer pairs for the construction of the Aa*VeA* knockout mutant

| Primers | Sequence (5´🡪3´) | Length (bp) | Annealing temperature (°C) |
| --- | --- | --- | --- |
| Aa*VeA*-up-F | ATGGATGTGCGGATGATG | 1458 | 54 |
| Aa*VeA*-up-R | caaaataggcattgatgtgttgacctccGATGCTGCGTCTCTATGG |  |  |
| Aa*VeA*-down-F | ctcgtccgagggcaaaggaatagagtagCCGTAGTAGGAATGGTCTTC | 1485 | 54 |
| Aa*VeA*-down-R | GGCTATGGCAATGAACTGA |  |  |
| *hph*-F | GGAGGTCAACACATCAATGCCTATT | 1349 | 54 |
| *hph*-R | CTACTCTATTCCTTTGCCCT |  |  |
| Aa*VeA*-knock-F | TTCTGGTGGATGGCGTAT | 3430 | 55 |
| Aa*VeA*-knock-R | ATGTGTTGAGCGAAGGAG |  |  |
| Aa*VeA*-out-F | ATACACCAGCGGACCTAC | 1823 | 55 |
| Aa*VeA*-out-R | CTGAAAGCACGAGATTCTTC |  |  |
| Aa*VeA*-in-F | GCAGTCTCCTCAACACTC | 476 | 55 |
| Aa*VeA*-in-R | GGTAAGAAGGCGACACAG |  |  |
| Aa*VeA*-qF | CGTGAGAACAAGTCGTCAA | 106 | 58 |
| Aa*VeA*-qR | TGGATGGTGGATGGTAGTT |  |  |
| *hph*-qF | GCATAACAGCGGTCATTG | 101 | 58 |
| *hph*-qR | CTCCATACAAGCCAACCA |  |  |
| β-tubulin-F | TCATACTTCGTTGAGTGGAT | 111 | 58 |
| β-tubulin-R | CTGGATGGAGGTGGAGTT |  |  |


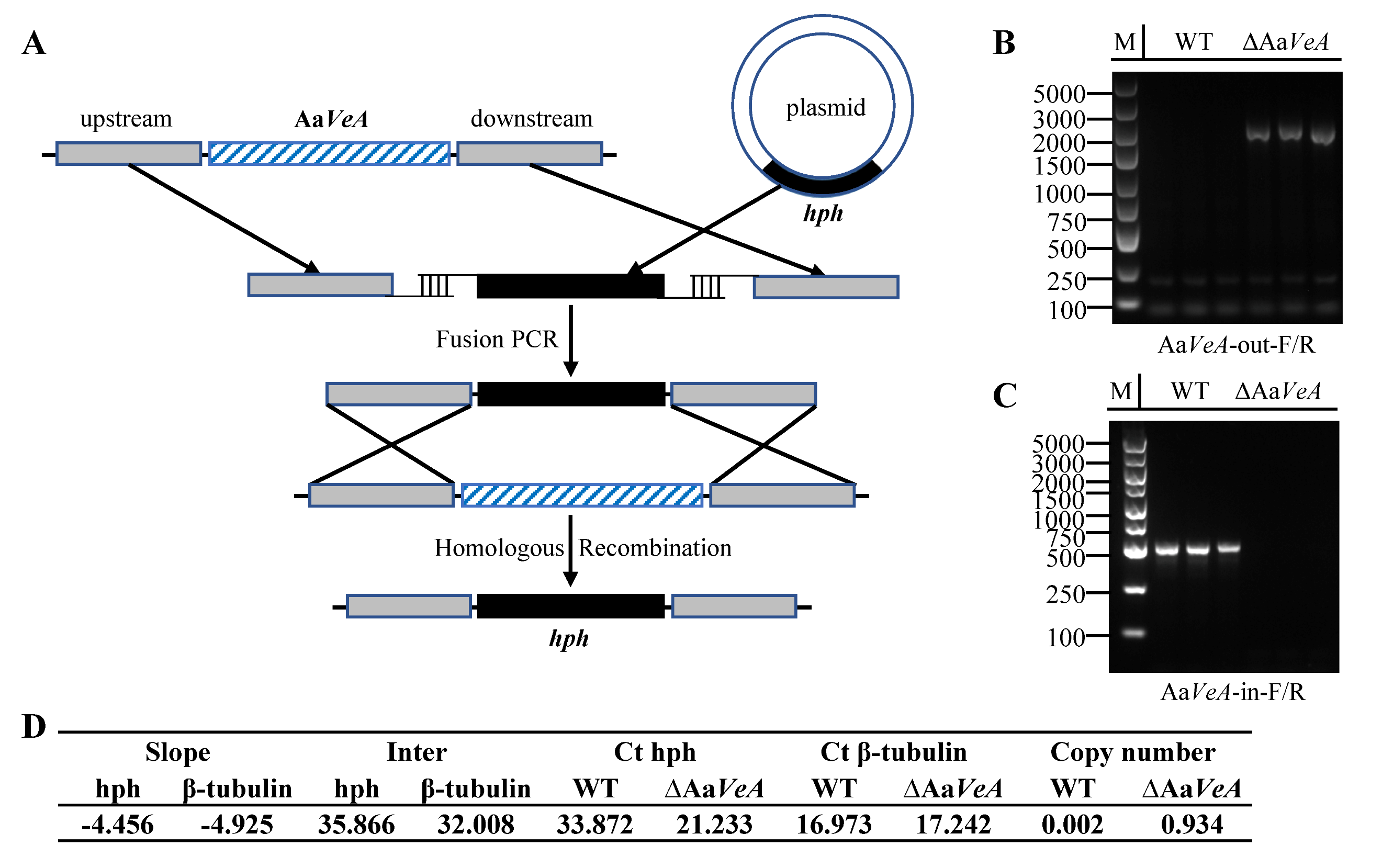


**Supplementary Figure S1** Aa*VeA* gene disruption and the identification of the mutant in *A. alternata*. (A) Schematic diagram of AaVeA gene disruption based on homologous recombination. (B) PCR identification for amplification region flanking from the upstream sequence of Aa*VeA* to the partial sequence of *hph*. (C) PCR amplification analysis of partial Aa*VeA* sequence. (D) The copy number expectation of *hph* gene integrated in the genome of the mutant based on the reference of Zhang et al (2016).

**Supplementary Table S2** Primer pairs for the relative level of gene expression in *A. alternata*

| Primers | Sequence (5´🡪3´) | Length (bp) | Annealing temperature (°C) |
| --- | --- | --- | --- |
| TeA-NRPS-cF | TCAGGTCATCCAGACATTAG | 102 | 58 |
| TeA-NRPS-cR | GAGTGAACAGCCATGAATG |  |  |
| TeA-TP-cF | TCAAGACCACGCATGATG | 92 | 58 |
| TeA-TP-cR | CAGACAACAGCCAGGATG |  |  |
| Aa*LaeA*-cF | CTACCAGTGGGACCTCAA | 102 | 58 |
| Aa*LaeA*-cR | CAGATGTGGATGTTGTTGTA |  |  |





**Supplementary Figure S2** The number of the significantly up-regulated and down-regulated differentially expressed genes (DEGs). The number of DEGs were statistically analyzed and displayed in histogram in comparison within two groups. As shown in the figure, the red bars represent the up-regulated DEGs, and the blue bars represent the down-regulated DEGs.

**Supplementary Table S3** The summary of raw RNA-seq data

| Sample | Raw Data (Reads) | Base | Valid Data (Reads) | Base | Valid Ratio(reads) | Q20% | Q30% | GC content% |
| --- | --- | --- | --- | --- | --- | --- | --- | --- |
| ΔAa*VeA*_D1 | 55390862 | 8.31G | 50595620 | 7.59G | 91.34 | 99.99 | 97.87 | 53.50 |
| ΔAa*VeA*_D2 | 54246834 | 8.14G | 48822692 | 7.32G | 90.00 | 99.99 | 97.92 | 53.50 |
| ΔAa*VeA*_D3 | 51873652 | 7.78G | 47107420 | 7.07G | 90.81 | 99.99 | 97.90 | 53.50 |
| ΔAa*VeA*_W1 | 51482536 | 7.72G | 48098330 | 7.21G | 93.43 | 99.99 | 97.86 | 54.00 |
| ΔAa*VeA*_W2 | 54155260 | 8.12G | 53209856 | 7.98G | 98.25 | 99.98 | 97.93 | 52.50 |
| ΔAa*VeA*_W3 | 51192702 | 7.68G | 46471476 | 6.97G | 90.78 | 99.99 | 98.10 | 53.50 |
| WT_D1 | 52516990 | 7.88G | 47667118 | 7.15G | 90.77 | 99.99 | 98.07 | 54.00 |
| WT_D2 | 49550778 | 7.43G | 44963398 | 6.74G | 90.74 | 99.99 | 97.99 | 53.50 |
| WT_D3 | 49923214 | 7.49G | 47341038 | 7.10G | 94.83 | 99.99 | 97.71 | 53.50 |
| WT_W1 | 49367736 | 7.41G | 45985048 | 6.90G | 93.15 | 99.99 | 98.25 | 53.50 |
| WT_W2 | 50765066 | 7.61G | 47857282 | 7.18G | 94.27 | 99.99 | 97.89 | 53.50 |
| WT_W3 | 52404278 | 7.86G | 48144564 | 7.22G | 91.87 | 99.99 | 98.06 | 54.00 |


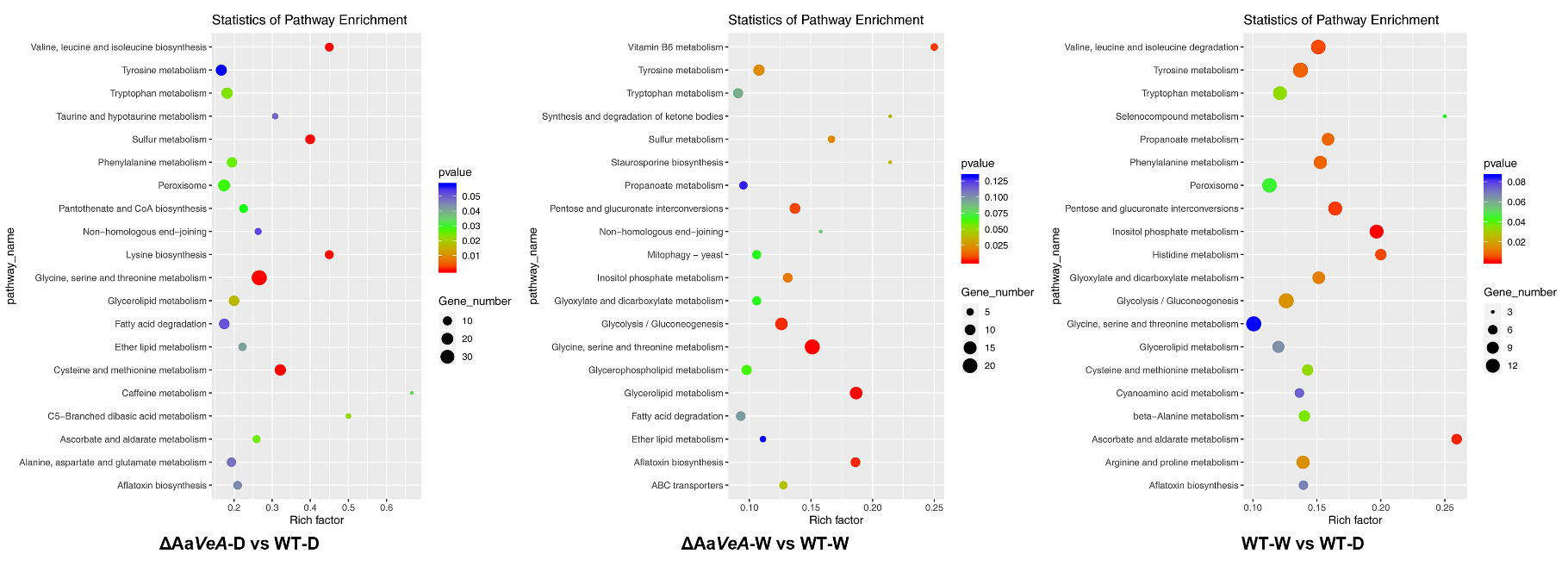


**Supplementary Figure S3** The enrichment analysis of Kyoto Encyclopedia of Genes and Genomes (KEGG) pathway in comparison within two groups. The comparison analysis includes the groups of ΔAa*VeA*-D vs WT-D, ΔAa*VeA*-W vs WT-W, and WT-W vs WT-D. Among them, “D” represents the treatment in darkness, and “W” represents the illumination treatment with white light.

**REFERENCES**

Zhang, J., Zhu, L., Chen, H., Li, M., Zhu, X., Gao, Q., et al. (2016). A polyketide synthase encoded by the gene An15g07920 is involved in the biosynthesis of ochratoxin A in *Aspergillus niger*. *J Agr Food Chem* 64, 9680–9688. doi:10.1021/acs.jafc.6b03907.
