# Supplementary figures and images for: Roles of AaVeA on Mycotoxin Production via Light in Alternaria alternata
Source: Front Microbiol. 2022 Feb 18;13:842268. doi: 10.3389/fmicb.2022.842268 (PMC8894881; doi:10.3389/fmicb.2022.842268)

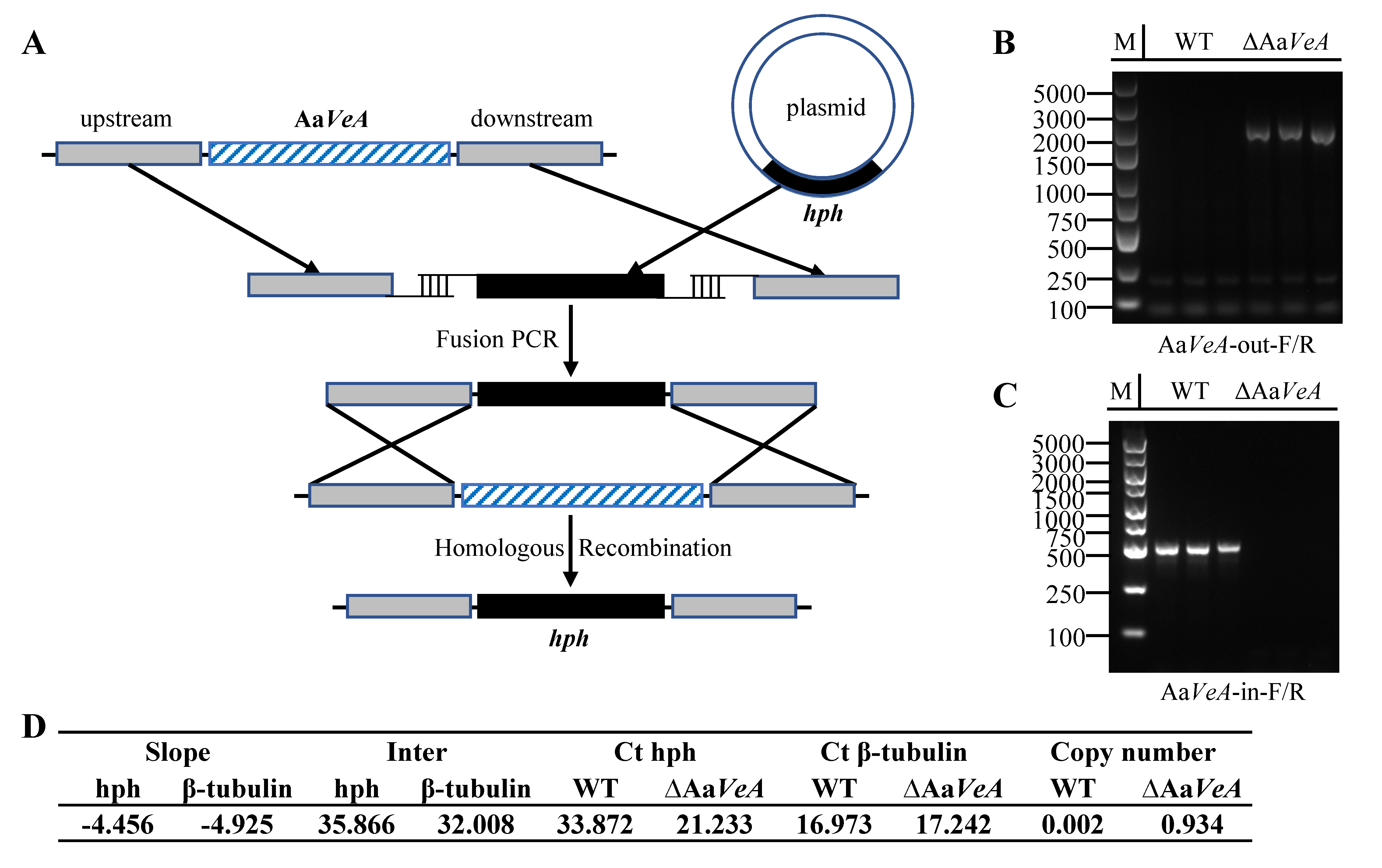

Supplement: Supplementary file 3 [file Image_1.TIF]

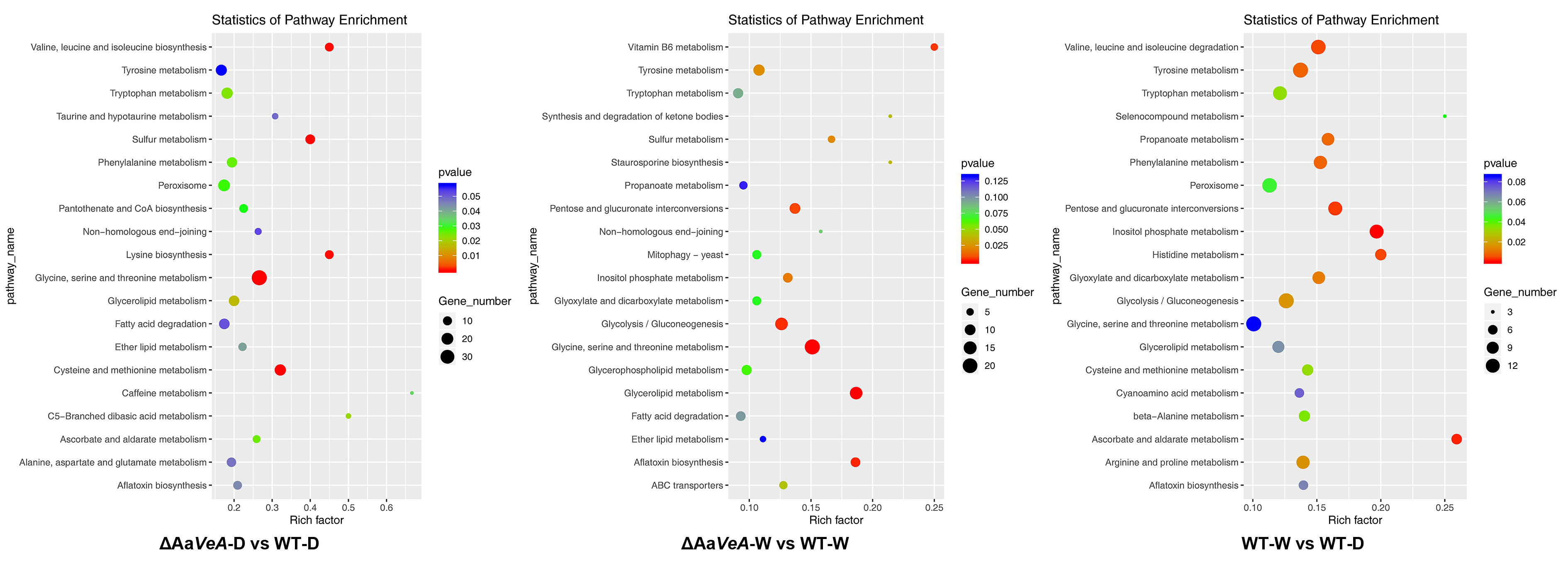

Supplement: Supplementary file 5 [file Image_3.TIF]
